# Psychometric Evaluation of the Performance Perfectionism Scale for Sport in the South Korean Context

**DOI:** 10.3390/bs15040424

**Published:** 2025-03-26

**Authors:** Yeongjun Seo, Hwasup Ko, Bumsoo Kim

**Affiliations:** 1 Department of Kinesiology, University of North Carolina at Greensboro, Greensboro, NC 27412, USA; y_seo4@uncg.edu; 2 Department of Physical Education, Kyunghee University, Yongin-si 17104, Republic of Korea; kimbam19@gmail.com

**Keywords:** confirmatory factor analysis, cultural validity, perfectionism, scale validation, student-athletes

## Abstract

The purpose of this study was to validate the Performance Perfectionism Scale for Sport (PPS-S) for use in South Korean student-athletes, addressing the critical need for a culturally appropriate measure of perfectionism in sport. The PPS-S was translated following established cross-cultural research protocols, including forward-backward translation and cognitive interviews. Participants were 332 collegiate athletes (79.5% male, 20.5% female; proportionate to the national collegiate athletic population distribution) registered with the Korean Sport and Olympic Committee. Confirmatory factor analysis using robust maximum likelihood estimation confirmed the three-factor structure (self-oriented, socially prescribed, and other-oriented perfectionism) with acceptable model fit indices (*χ*^2^[49] = 163.54, *p* < 0.001; CFI = 0.906; RMSEA = 0.084, 90% CI [0.071, 0.097]; SRMR = 0.077). This validation represents a significant advancement in South Korean sport psychology, providing practitioners and researchers with the first psychometrically sound instrument for assessing perfectionism in sport and informing culturally tailored interventions. It addresses the limitations of previous research that relied on general perfectionism measures, which compromised domain and cultural validity by potentially misrepresenting athletes’ perfectionistic tendencies. Future research is needed to examine how this PPS-S performs distinctively compared to traditional general perfectionism measures and investigate its associations with various psychological outcomes.

## 1. Introduction

South Korean society fosters a high-pressure environment where athletes, parents, and coaches exhibit little tolerance for mistakes or underperformance (Seo & Kim, 2022). This pressure stems from intense academic and athletic competition tied to university admissions and the limited opportunities for athletes to advance to professional sports (S.-Y. Kim, 2020). As a result, the way in which athletes are compelled to exert excessive effort to meet high expectations reinforces the development of perfectionistic tendencies that can lead to maladaptive psychosocial outcomes (Dunn et al., 2005; Hill et al., 2018). The culture of sport in South Korea is deeply rooted in a performance-oriented and outcome-centered ideology, which extends beyond the sport realm to influence society as a whole. Particularly, the tendency to equate success solely with achievements (e.g., number of medals) undermines sport ethics and fundamental human rights by prioritizing performance outcomes over the well-being and dignity of athletes (Nam et al., 2018).

Parents and coaches play a significant role in developing perfectionistic tendencies within South Korea’s elite sport culture (Seo & Kim, 2022). Parents often link their children’s athletic achievements to their own sense of success, while coaches focus on team performance by imposing rigorous standards, collectively placing excessive psychological burdens on athletes (B.-S. Kim, 2021). This intense focus on achievement underscores key cultural distinctions between Eastern and Western approaches to sport and athlete development. While athletes from Western cultures typically emphasize self-enhancement in sport, athletes from Eastern contexts prioritize continuous self-improvement, rooted in cultural tendencies toward objective self-evaluation (Yasuda & Masuda, 2024). These pressures may uniquely shape how perfectionism manifests among South Korean student-athletes, highlighting the need for culturally sensitive approaches to understanding and addressing their psychological needs.

Despite the critical role perfectionism plays in shaping athletes’ overall health, performance, and well-being (Hill et al., 2018; Schellenberg et al., 2025; Yang et al., 2023), there is currently no psychometrically validated tool specifically designed to assess perfectionism within the South Korean sport context. Instead, researchers have predominantly relied on the translated version of Hewitt and Flett’s (1991) general perfectionism scale to evaluate athletes’ perfectionistic tendencies (e.g., S.-K. Kim & Oh, 2008; D.-H. Kim & Park, 2019; Y.-H. Kwon, 2024). However, this measure was neither developed nor validated for sport-specific contexts, raising concerns about their construct and cultural validity when applied to athletes (Hill et al., 2016). This gap in culturally and contextually appropriate assessment tools underscores the importance of exploring existing measures and their applicability to South Korean athletes, particularly within performance-driven environments.

### 1.1. Measures of Perfectionism

To date, numerous measures of perfectionism have been developed, and sport-specific measures have been proposed in various ways by different researchers, leading to confusion about which measure is most appropriate for research and practice (Madigan, 2023). Early research initially conceptualized perfectionism as a unidimensional and maladaptive construct (Burns, 1980). Building on this initial conceptualization, various measures were developed and implemented, leading to increasingly sophisticated conceptualizations of the construct. Research in the early 1990s sought to understand and evaluate perfectionism as a multidimensional construct, leading to the development of multidimensional perfectionism measures and generating substantial empirical investigation of the construct (Frost et al., 1990; Hewitt & Flett, 1991). As the conceptualization and measurement of perfectionism for the general population evolved, the 2000s marked an increased effort to validate these constructs within the sport context (e.g., Gotwals & Dunn, 2009; Hill et al., 2016; Stoeber et al., 2006). The need to validate the assessment of perfectionistic tendencies tailored to the sport context arises from the fact that athletes, dancers, and exercisers often maintain elevated performance standards within their respective domains, which can undermine the validity of general psychological measures in capturing individual differences in these performance-driven settings (Madigan, 2023).

The Performance Perfectionism Scale-Sport (PPS-S; Hill et al., 2016) is one such example, adapting Hewitt and Flett’s (1991) multidimensional perfectionism framework to the sport context. The scale consists of three subscales measuring self-oriented, socially prescribed, and other-oriented perfectionism, each comprising four items rated on a 5-point Likert scale. Self-oriented perfectionism entails setting unrealistically high self-standards and engaging in rigorous self-evaluation, socially prescribed perfectionism reflects perceived external expectations of perfection, and other-oriented perfectionism involves imposing such demands on others (Flett & Hewitt, 2005; Hewitt & Flett, 1991; Hill et al., 2016). The dimensions of perfectionism have distinct implications for athletes. While socially prescribed and other-oriented perfectionism are generally associated with maladaptive outcomes (e.g., competitive anxiety, burnout, internalized shame), self-oriented perfectionism demonstrates potential adaptive benefits (Flett & Hewitt, 2005; Ko, 2023; W. Kwon & Cho, 2020; Yang et al., 2023). Self-oriented perfectionism can act as a protective factor against burnout, particularly among high-performing athletes who possess strong coping resources and achieve competitive success (Seo & Kim, 2022; Yang et al., 2023). However, despite the potential benefits of self-oriented perfectionism, research demonstrates that all dimensions of perfectionism can trigger significant psychological stress, particularly sports-related stress for athletes (D.-M. Kim & Cho, 2023).

Multiple studies have adapted and psychometrically evaluated the PPS-S across various international contexts, including Turkey, the United Kingdom, and Brazil (Angelo et al., 2019; Esentaş et al., 2020; Olsson et al., 2022). While some variations exist in internal consistency and the number of retained items, the three-factor structure has remained stable across cultures. Reported reliability estimates for the subscales range from 0.65 to 0.83 (Angelo et al., 2019; Olsson et al., 2022). The need for culturally sensitive and contextually relevant perfectionism scales is particularly important in South Korean sport culture, where traditional emphasis on age-based respect and seniority influences how athletes perceive and interact with others. For example, research using the Multidimensional Inventory of Perfectionism in Sport (MIPS; Stoeber et al., 2006) found that Western concepts like “teammate” fail to capture the nuanced distinctions between senior members, peers, and junior members that are central to South Korean sport settings (Seo & Kim, 2022). This underscores the importance of developing measurement tools that reflect the unique interpersonal dynamics of the South Korean context. The PPS-S offers a distinct advantage in cross-cultural application compared to other perfectionism measures in sport. By instructing respondents to consider relationships they perceive as relevant (Hill et al., 2016) when responding to questions about their expectations of others or the expectations placed on them by others (i.e., specifically those from whom they expect perfectionism or who prescribe perfectionistic standards), the PPS-S allows athletes to interpret “people” and “others” based on their own social position. This flexibility makes the measure more personal and adaptable to individual and cultural contexts, enhancing its potential utility for South Korean student-athletes.

### 1.2. Present Study

The purpose of the present study was to validate the Performance Perfectionism Scale-Sport (PPS-S; Hill et al., 2016) in a sample of South Korean student-athletes. Given that South Korean researchers have primarily relied on Hewitt and Flett’s (1991) general population measure to assess athletes’ perfectionistic tendencies, the PPS-S (Hill et al., 2016) emerges as a promising candidate for addressing the need for a sport-specific perfectionism measure that is both culturally relevant and psychometrically sound. Psychometric validation studies that ensure cultural validity are an essential and inevitable step in advancing sport psychology research and practice in South Korea (Y. Kim et al., 2021). Given that competitive athletes across international settings share a common focus on performance optimization, athletic excellence, and achievement (McKinney et al., 2019), we hypothesize that the PPS-S will demonstrate robust psychometric validity in South Korean competitive student-athletes. Furthermore, consistent with findings from D.-M. Kim and Cho (2023), each dimension of perfectionism (self-oriented, socially prescribed, and other-oriented) will show a significant positive relationship with perceived stress. This measurement will, in turn, support the development of culturally tailored, evidence-based interventions and strategies aimed at promoting South Korean athletes’ psychosocial health, well-being, and performance.

## 2. Methods

### 2.1. Translation Process

The translation and back-translation process followed established protocols for cross-cultural research (Brislin, 1970; Jones et al., 2001). Initially, two bilingual experts independently translated the instrument from English to Korean, followed by blind back-translations to English by two different experts. The translated versions were then reviewed in collaborative meetings between three members of the research team and three bilingual experts who held or were pursuing Ph.D. degrees in Counseling, Linguistics, and Kinesiology with proficiency in both Korean and English. These meetings focused on resolving discrepancies and refining cultural nuances, with additional back-translations and reviews conducted until consensus was achieved. Following the translation process, the translated questionnaire underwent field testing through cognitive interviews, where student-athlete participants (*n* = 10) verbalized their thought processes while answering questions to ensure content and face validity (Boateng et al., 2018). The original English questionnaire and its Korean translation are presented in Table 1.

### 2.2. Procedures

The research team recruited participants through established connections within university athletic programs. Initial contact was made with university athletic team coaches to explain the study’s purpose and request their assistance in facilitating student-athlete participation. Student-athletes were eligible for inclusion if they were enrolled in university and registered with the Korean Sport and Olympic Committee. Data collection proceeded through two methods. Among coaches who agreed to participate, research team members visited their teams to administer the survey in person. To minimize potential response bias, coaches and other sport personnel were separated from student-athletes during survey completion. For teams where coaches indicated their athletes could not participate in person due to practice schedules or competition conflicts, an alternative online survey was provided through Google Forms, with survey links distributed by their respective coaches. Student-athlete participants were required to provide informed consent before accessing the survey questions. The survey took approximately 15 min to complete. Data collection occurred between December 2024 and January 2025. Study procedures and ethical considerations were reviewed and approved by the Institutional Review Board of a private university in South Korea.

### 2.3. Measures

The demographic questionnaire (i.e., academic year, years of sport career, national team experience, sex, sport type) was included at the beginning of the survey, followed by measures of perfectionism. Perceived stress measure was included to assess convergent validity. In addition to the measures reported here (PPS-S and PSS), other psychological measures were collected as part of a broader research initiative. However, these additional measures were not included in the current study as they are not directly relevant to the research questions and hypotheses.

#### 2.3.1. Perfectionism Scale for Sport

The Korean-translated version of the Performance Perfectionism Scale for Sport (PPS-S; Hill et al., 2016) was used to assess perfectionistic tendencies. This instrument adapted Hewitt and Flett’s (1991) model of perfectionism to the sport context. The PPS-S consists of three subscales measuring self-oriented (e.g., I am tough on myself when I do not perform perfectly), socially prescribed (e.g., People always expect more, no matter how well I perform), and other-oriented performance perfectionism (e.g., I have a lower opinion of others when they do not perform perfectly), capturing the core features of Hewitt and Flett’s dimensions while focusing on performance rather than general life domains. Each item was rated on a 5-point Likert scale, ranging from 1 (*strongly disagree*) to 5 (*strongly agree*).

#### 2.3.2. Perceived Stress

The Perceived Stress Scale (PSS; Cohen et al., 1983) is a 10-item measure designed to assess stress appraisal, including perceptions of control, overload, and emotional responses to stressful experiences. The PSS was later translated into Korean and validated for use within the South Korean context (J. Park & Seo, 2010). An example item is, “In the last month, how often have you felt nervous and ‘stressed’?” Participants responded using a 5-point Likert scale ranging from 0 (never) to 4 (very often). The omega coefficient for the PSS in this study was 0.83.

### 2.4. Data Management and Analysis

Data management and analyses were conducted using the *lavaan* package version 0.6–19 (Rosseel, 2012) in R (version 4.4.2; R Core Team, 2024). To ensure reproducibility, the data and code used in this study have been published and are available at https://doi.org/10.17605/OSF.IO/WV2QA (accessed on 24 January 2025). Among the final 332 cases, no missing values were identified across the 12 PPS-S items. Descriptive statistics were examined for all items, with means ranging from 2.09 to 3.47 (SD 0.91–1.18) across different dimensions of perfectionism (Table 2). Normality and outliers were inspected following Kline’s (2023) guidelines (i.e., skewness < 3 and kurtosis < 8). All items demonstrated acceptable levels of skewness (−0.51, 0.69) and kurtosis (−0.90, 0.10). Outliers were examined using Mahalanobis distance, which identified nine cases as potential outliers. However, the differences were minimal, and to preserve the natural variance in the personality data, these cases were retained (Bandalos & Finney, 2018).

Confirmatory factor analysis (CFA) was used to validate the three-factor structure of the Korean version of the PPS-S. Several fit indices were consulted to evaluate model fit. CFA is a latent variable modeling technique that evaluates how well-observed variables represent underlying theoretical constructs (Kline, 2023). The first indicator for each latent factor was fixed to 1, allowing all other factor loadings to be estimated relative to this reference item. Several fit indices were consulted to evaluate model fit. Although a non-significant chi-square test indicates a good model fit, this index is sensitive to sample size (Kline, 2023). Thus, additional indices were examined based on established guidelines. According to Hu and Bentler (1999), comparative fit index (CFI) values above 0.90 and standardized root mean squared residual (SRMR) values below 0.08 indicate acceptable model fit. Additionally, MacCallum et al. (1996) suggested that the root mean square error of approximation (RMSEA) values between 0.05 and 0.10 indicate fair fit, with values below 0.08 representing good fit. Based on established guidelines, our sample size was sufficient for conducting CFA (Kline, 2023). Following the validation, the convergent validity of each perfectionism dimension was assessed by examining its correlation with perceived stress using Pearson correlation analysis.

## 3. Result

The final sample included 68 female student-athletes (20.5%) and 264 male student-athletes (79.5%), reflecting a sex distribution consistent with the broader collegiate athletic population, where female student-athletes constitute approximately 20% (Korean Sport & Olympic Committee, 2024). There were 18 different sports represented in the sample, with the majority of participants being freshmen (*n* = 116, 34.9%) and sophomores (*n* = 125, 37.7%). Participants, on average, had 9.32 ± 3.14 (mean ± standard deviations) years of competitive experience in their respective sport, with 26.5% either playing or having played for the national team. Detailed descriptive information is presented in Table 3.

### 3.1. Confirmatory Factor Analysis

**Figure 1:**
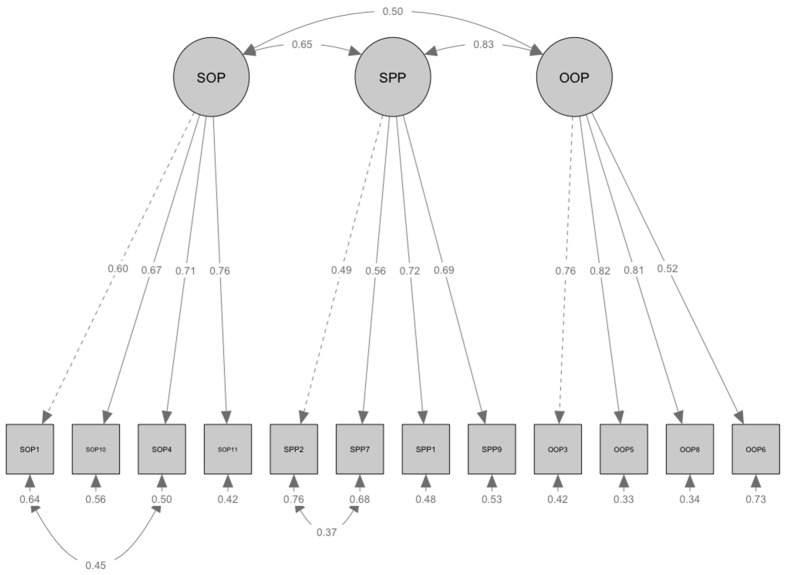
Results of Confirmatory Factor Analysis. *Note*. N = 332. All estimates presented in the figure are standardized. χ^2^(49) = 163.54, *p* < 0.001; CFI = 0.906, RMSEA = 0.084, 90% CI [0.071, 0.097], SRMR = 0.077. SOP = Self-Oriented Perfectionism; SPP = Socially Prescribed Perfectionism; OOP = Other-Oriented Perfectionism.

Examination of Mardia’s multivariate normality test indicated significant deviations from multivariate normality (*p* < 0.001); therefore, robust maximum likelihood (MLR) estimation was employed. The initial model exhibited poor fit to the data based on scaled fit indices: χ^2^(51) = 224.01 (*p* < 0.001), CFI = 0.858, RMSEA = 0.101, 90% CI [0.089, 0.113], SRMR = 0.084. All indices fell outside the recommended thresholds for acceptable model fit. Examination of the modification indices suggested that specifying residual correlations between SOP1 and SOP4, as well as SPP2 and SPP7, could improve model fit. These modifications were theoretically justifiable due to shared method variance arising from similar item content and wording. After implementing these modifications, the revised model showed an acceptable fit across multiple indices: CFI = 0.906, RMSEA = 0.084, 90% CI [0.071, 0.097], SRMR = 0.077, although the chi-square test remained significant, χ^2^(49) = 163.54, (*p* < 0.001). The comprehensive evaluation of fit indices suggests that the revised model provides an acceptable fit to the data. The standardized factor loadings demonstrated adequate psychometric properties across the three dimensions (i.e., SOP: λ = 0.60–0.76; SPP: λ = 0.49–0.72; OOP: λ = 0.52–0.82). The latent factor correlations indicated moderate to strong relationships between dimensions of perfectionism (rs = 0.50–0.83). Each subscale had omega coefficients of 0.84, 0.79, and 0.84 for self-oriented, socially prescribed, and other-oriented perfectionism, respectively (see Figure 1).

### 3.2. Convergent Validity

Convergent validity between the three dimensions of perfectionism (self-oriented, socially prescribed, and other-oriented) and perceived stress was established through Pearson correlation analyses (Table 4). Results revealed significant positive correlations between all perfectionism dimensions and stress (*r* ranging from 0.341 to 0.440; *p* < 0.001), providing support for the theoretical relationship between dimensions of perfectionism and perceived stress.

## 4. Discussion

The present study aimed to validate the PPS-S in the South Korean context, addressing a critical gap in culturally appropriate assessment tools for perfectionism in sport. The results provide preliminary support for the three-factor structure of the Korean version of the PPS-S, though some modifications were necessary to achieve an acceptable model fit. These findings offer important insights into both the measurement of perfectionism in South Korean sports and the cross-cultural applicability of the PPS-S.

The overall goodness of fit statistics showed a similar pattern to Hill et al.’s (2016) validation across different samples. Yet, the residual correlation between SOP items 1 (“I am tough on myself when I do not perform perfectly”) and 4 (“I put pressure on myself to perform perfectly”) likely reflects their shared focus on self-imposed performance pressure, suggesting these items capture closely related aspects of self-critical perfectionism. Similarly, the correlation between SPP items 2 (“People always expect more, no matter how well I perform”) and 7 (“People always expect my performances to be perfect”) indicates overlapping content in measuring perceived external expectations, particularly through their shared emphasis on the persistent nature of others’ expectations (“always expect”). These item-level relationships suggest that while the items contribute to their respective factors, they may represent closely related manifestations of their underlying constructs in the South Korean context. Future research should examine whether these residual correlations are unique to the South Korean context or represent a universal feature of performance perfectionism measurement. Cross-cultural validation studies could help determine if the relationship between self-oriented and socially prescribed items varies across cultural contexts.

Although the moderate to strong correlations between dimensions align broadly with Western findings, the strong association between other-oriented and socially prescribed perfectionism might suggest these dimensions measure similar constructs in the South Korean context. This relationship is particularly noteworthy as both dimensions are typically associated with maladaptive outcomes (Flett & Hewitt, 2005; W. Kwon & Cho, 2020; Yang et al., 2023), though they remain theoretically different. In contrast, self-oriented perfectionism demonstrated more distinct functioning, aligning with previous research suggesting its potential for both adaptive and maladaptive outcomes depending on context (Flett & Hewitt, 2005; Yang et al., 2023).

All dimensions of perfectionism showed significant positive correlations with perceived stress. These findings suggest that while self-oriented perfectionism may have adaptive potential, it can still contribute to stress when unrealistic self-standards become overwhelming (D.-M. Kim & Cho, 2023). Both socially prescribed and other-oriented perfectionism also demonstrated associations with stress, reinforcing their links to maladaptive psychological outcomes. Future research is warranted to examine these dimensional relationships with additional outcome variables (e.g., performance anxiety, athletic burnout, competitive achievement) in South Korean athletes.

International evidence comparing Eastern and Western cultures has highlighted differences in how individuals perceive themselves and their abilities. In Western cultures, people often evaluate their abilities positively, emphasizing strengths and talents (i.e., self-enhancement), whereas, in Eastern cultures, individuals tend to focus more on their weaknesses and show a strong desire for self-improvement (Yasuda & Masuda, 2024). This pattern is also evident in the South Korean context, where high expectations from parents and coaches (Seo & Kim, 2022), combined with athletes’ high achievement orientation, often lead to dissatisfaction and shame (Ko, 2023), manifesting as dichotomous thinking in which minor mistakes or failures are perceived as complete failures (Egan et al., 2007). This culturally driven focus on achievement, with its emphasis on performance enhancement, can interact with maladaptive perfectionism, resulting in negative psychological outcomes such as anxiety and stress while potentially undermining athletic performance (B.-S. Kim, 2021). Thus, the emphasis on performance enhancement in sport psychology has led to the widespread use of psychological skills training, which improves factors like team cohesion and confidence and, when combined with physical and technical training, enhances athletes’ overall performance (B.-J. Kim et al., 2020; Yoo & Cho, 2024). However, the interplay between cultural influences, interpersonal relationships, and structured interventions remains underexplored, particularly in how different sport contexts shape perfectionistic tendencies.

The influence of perfectionism varies based on the nature of the sport. In individual sports, where outcomes are directly tied to personal effort, perfectionistic tendencies are primarily driven by self-imposed expectations or externally imposed standards from coaches and parents (Son et al., 2024). In contrast, team sports in South Korea operate within a culturally ingrained hierarchy, where younger athletes defer to older teammates. This hierarchical system introduces unique interpersonal stressors, fostering perfectionistic demands on others, an aspect that has been relatively underexplored compared to other dimensions of perfectionism. Seo and Kim (2022) further highlighted developmental differences in perfectionism, noting that middle school athletes newly entering elite sports experience the highest levels of perceived pressure from coaches and parents, contributing to the development of maladaptive perfectionism. Although this pressure tends to show a diminishing pattern as athletes transition to high school and college, longitudinal studies are needed to examine how multidimensional perfectionistic tendencies evolve across developmental stages.

The findings of this study highlight the necessity of expanding the applicability of validated perfectionism scales within sports contexts. To achieve this, it is critical to standardize these scales, thereby enabling sports psychology consultants (Y. Kim et al., 2021) to systematically measure and evaluate athletes’ perfectionistic tendencies in sports settings. Such standardization would facilitate the quantitative assessment of athletes’ psychological characteristics and enhance their use in counseling and psychological support interventions. To support these efforts, collaboration with key sport organizations and governmental bodies (i.e., Korea Sport Science Institute, Korea University Sport Federation, and the Korean Sport and Olympic Committee) is needed. Establishing a systematic framework for the policy-driven application of validated scales will enable the development and implementation of culturally tailored psychological support policies, ensuring the effective integration of perfectionism scales within sport settings.

## 5. Strength and Limitations

A key strength of the PPS-S in the South Korean context is its adaptable approach to interpersonal relationships. Unlike other sport perfectionism measures that use specific terms like “teammate” (Stoeber et al., 2006), the PPS-S enables respondents to reflect on relationships they deem relevant when answering items. This adaptability is particularly important in accommodating the hierarchical nature of South Korean sports culture, where age-based respect and seniority heavily shape athletic relationships. While this study did not specifically address the complexities of these dynamics, future research should explore how the influence of expectations, whether imposed on or by others, affects social interactions and leads to differing outcomes in different dimensions of perfectionism (Seo & Kim, 2022). This is a critical consideration for both practitioners and researchers, as these cultural dynamics influence how perfectionism manifests in South Korean athletes, emphasizing the need for measurement tools that are both psychometrically sound and culturally attuned to these unique interpersonal dynamics.

Despite these strengths, several limitations warrant consideration. First, while the proportion of female athletes in our sample reflects the broader collegiate athletic population in South Korea, the unequal gender distribution limited our ability to examine measurement invariance between male and female athletes. Future research should explore both gender-based and temporal measurement invariance to further strengthen the psychometric evidence for the PPS-S. Furthermore, the validation was limited to collegiate student-athletes, leaving a critical gap in understanding younger student-athletes (i.e., junior high and high school athletes). In South Korea, these younger athletes often face heightened external pressures from coaches and parents, which fosters the development of maladaptive perfectionistic tendencies linked to burnout (Seo & Kim, 2022). Expanding validation efforts to include younger athletes would provide insights into the development of perfectionism across different stages of athletic careers.

## 6. Conclusions

This study validated the PPS-S as a culturally relevant and psychometrically sound tool for assessing perfectionism among South Korean student-athletes. By addressing the cultural and contextual nuances of perfectionism, the PPS-S offers a valuable psychometric tool for researchers and practitioners aiming to promote athletes’ psychological health, well-being, and performance in South Korea. Future work should leverage this tool to enhance understanding and support for athletes in high-pressure, performance-driven environments.

## Figures and Tables

**Table 1:** Translation of the Questionnaire.

Item	Dimension	English	Korean
1	SOP	I am tough on myself when I do not perform perfectly.	내가 완벽하게 수행하지 못할 때, 나는 나 자신에게 가혹하다.
4	SOP	I put pressure on myself to perform perfectly.	나는 완벽하게 수행하기 위해 스스로에게 압박을 준다.
10	SOP	I only think positively about myself when I perform perfectly.	나는 완벽하게 수행할 때만 나 자신에 대해 긍정적으로 생각한다.
11	SOP	To achieve the standards I have for myself, I need to perform perfectly.	내가 정한 기준에 도달하기 위해 나는 완벽하게 수행해야 한다.
2	SPP	People always expect more, no matter how well I perform.	내가 아무리 좋은 퍼포먼스를 보여도 사람들은 항상 더 높은 기대를 한다.
7	SPP	People always expect my performances to be perfect.	사람들은 항상 내가 완벽하게 수행하길 기대한다.
9	SPP	People view even my best performances negatively.	사람들은 내 최선의 퍼포먼스도 부정적으로 본다.
12	SPP	People criticize me if I do not perform perfectly.	내가 완벽하게 수행하지 않으면 사람들은 나를 비판한다.
3	OPP	I have a lower opinion of others when they do not perform perfectly.	다른 사람들이 완벽하게 수행하지 않으면 나는 그들에 대해 낮게 평가한다.
6	OPP	I am never satisfied with the performances of others.	나는 다른 사람들의 퍼포먼스에 절대 만족하지 않는다.
8	OPP	I criticize people if they do not perform perfectly.	사람들이 완벽하게 수행하지 않으면 나는 그들을 비판한다.
5	OPP	I think negatively of people when they do not perform perfectly.	사람들이 완벽하게 수행하지 않으면 나는 그들에 대해 부정적으로 생각한다.

**Table 2:** Descriptive Statistics of Individual Items.

Item	Mean	SD	Skewness	Kurtosis
SOP1	3.27	1.004	−0.348	−0.342
SPP2	3.10	0.964	0.146	−0.380
OOP3	2.50	0.988	0.292	−0.476
SOP4	3.28	1.050	−0.312	−0.531
OOP5	2.26	0.983	0.460	−0.525
OOP6	2.59	1.055	0.366	−0.524
SPP7	3.01	1.013	−0.112	−0.526
OOP8	2.09	0.914	0.686	0.104
SPP9	2.19	0.998	0.633	−0.235
SOP10	3.00	1.176	0.000	−0.903
SOP11	3.47	1.052	−0.507	−0.182
SPP12	2.36	1.017	0.545	−0.252

*Note.* N = 332. SD = Standard Deviation. SOP = Self-Oriented Perfectionism; SPP = Socially Prescribed Perfectionism; OOP = Other-Oriented Perfectionism.

**Table 3:** Descriptive Statistics.

Characteristic	Frequency (%)
Sex	
Male	264 (79.5)
Female	68 (20.5)
Academic Year	
Freshman	116 (34.9)
Sophomore	125 (37.7)
Junior	72 (21.7)
Senior	19 (5.7)
National Team Status ^1^	
No National Team Experience	244 (73.5)
Previous Experience with the National Team	80 (24.1)
Current National Team Member	8 (2.4)
Sport Type	
Taekwondo ^2^	116 (34.9)
Soccer	61 (18.4)
Basketball	26 (7.8)
Baseball	25 (7.5)
Rugby	21 (6.3)
Bowling	12 (3.6)
Handball	11 (3.3)
Ice Hockey	10 (3.0)
Volleyball	9 (2.7)
Horseback Riding	9 (2.7)
Gymnastics	9 (2.7)
Ssireum ^3^	8 (2.4)
Archery	8 (2.4)
Kendo	2 (0.6)
Skiing	2 (0.6)
Badminton	1 (0.3)
Aerobics	1 (0.3)
Jiu-jitsu	1 (0.3)

*Note.* N = 332. ^1^ National representative team (e.g., university national team, Olympic team, Asian Games national team, U18, U20, or other national representative teams). ^2^ Includes Taekwondo demonstration. ^3^ Traditional Korean wrestling.

**Table 4:** Correlation Matrix of Perfectionism Dimensions and Stress.

Variable	SOP	SPP	OOP	Stress
SOP	3.25			
SPP	0.55	2.67		
OOP	0.44	0.62	2.36	
Stress	0.44	0.39	0.34	3.06

*Note.* SOP = Self-Oriented Perfectionism; SPP = Socially Prescribed Perfectionism; OOP = Other-Oriented Perfectionism. All values in the diagonal represent mean scores for each construct. All correlations are statistically significant at *p* < 0.001.

## Data Availability

The deidentified dataset, analysis code in R, and complete codebook used in this study are publicly available at: https://doi.org/10.17605/OSF.IO/WV2QA (accessed on 24 January 2025).
